# Melatonin in ovarian tissue preservation: from molecular mechanisms to clinical translation

**DOI:** 10.1590/1806-9282.7210EDIT

**Published:** 2026-11-02

**Authors:** Karla Krislane Alves Costa Monteiro, Edmund Chada Baracat, José Maria Soares

**Affiliations:** 1 Laboratório de Investigação em Ginecologia Molecular e Estrutural da Disciplina de Ginecologia, Departamento de Obstetrícia e Ginecologia, Faculdade de Medicina da Universidade de São Paulo – São Paulo (SP), Brazil.

Ovarian tissue cryopreservation and transplantation represent the only fertility preservation strategy available for prepubertal cancer patients and women requiring urgent gonadotoxic therapy. However, cryoinjury, ischemia-reperfusion damage, and oxidative stress significantly compromise follicular viability and graft longevity. Melatonin, a multifunctional indoleamine with potent antioxidant properties, has emerged as a promising adjunct to mitigate these challenges.

In a recently published systematic review, Monteiro et al.^1^ comprehensively evaluated the antioxidant actions of melatonin in animal models of ovarian tissue preservation. The analysis revealed that melatonin consistently reduces oxidative stress markers and enhances the expression and activity of key antioxidant enzymes across multiple experimental models. The systematic synthesis demonstrated that melatonin’s efficacy is dose-dependent and varies according to administration route, with intraperitoneal and subcutaneous routes showing particularly robust effects. Importantly, the review identified that melatonin’s antioxidant actions extend beyond direct radical scavenging to include the modulation of nuclear factor erythroid 2-related factor 2 (Nrf2)-mediated signaling pathways and the preservation of mitochondrial function^1^.

Oxidative stress represents a central pathophysiological mechanism in ovarian tissue injury during cryopreservation and transplantation. The generation of reactive oxygen species (ROS) during freeze-thaw cycles and subsequent reperfusion triggers lipid peroxidation, mitochondrial dysfunction, and DNA damage in granulosa cells and oocytes^2^. Melatonin exerts potent antioxidative effects through multiple pathways, significantly reducing ROS concentrations and improving follicular morphological integrity^3^.

A pivotal contribution from our group demonstrated that melatonin increases superoxide dismutase 2 (SOD2) levels and improves rat ovarian graft function after transplantation^4^. In this study, female rats underwent bilateral ovariectomy followed by heterotopic transplantation of frozen-thawed ovarian tissue. Melatonin was administered intraperitoneally at 10 mg/kg for 21 days post-transplantation. The results were striking: melatonin-treated animals exhibited significantly higher SOD2 immunoexpression in theca interna cells and interstitial cells, accompanied by reduced follicle-stimulating hormone (FSH) levels and favorable FSH/anti-Müllerian hormone ratios. Histological analysis revealed that melatonin-treated grafts contained more mature follicles, showed enhanced type I collagen deposition, and demonstrated improved angiogenesis compared to controls^4^. These findings are clinically significant because SOD2, the mitochondrial isoform of superoxide dismutase, serves as the primary defense against mitochondrial superoxide radicals. The preservation of SOD2 activity is crucial for maintaining mitochondrial homeostasis and preventing the oxidative damage that compromises granulosa cell function and oocyte quality.

Expanding upon these findings, our subsequent investigation examined the effects of melatonin on glutathione peroxidase (GPx) activity and overall antioxidant defense in cryopreserved ovarian transplants55. Melatonin treatment significantly increased GPx activity in grafted ovarian tissues, accompanied by reduced malondialdehyde levels, indicating decreased lipid peroxidation. Furthermore, melatonin upregulated the expression of GPx4, the selenoenzyme isoform critical for protecting cells against ferroptosis—an iron-dependent, oxidative stress-driven form of cell death^5^. The enhancement of GPx activity represents a complementary mechanism to SOD2 upregulation, as both enzymes work sequentially to neutralize ROS: SOD2 converts superoxide radicals to hydrogen peroxide, which is subsequently detoxified by GPx to water. This coordinated antioxidant defense is essential for maintaining the redox balance in grafted ovarian tissues, where ischemia-reperfusion injury creates a burst of oxidative stress.

A particularly innovative contribution from our group explored the use of scaffold-based melatonin delivery systems for frozen-thawed ovarian autografts^6^. This study represents a significant translational advance, moving beyond systemic melatonin administration toward localized, sustained delivery directly to the graft site. Using Gelfoam^®^ as a biodegradable scaffold loaded with melatonin, the researchers demonstrated that local delivery effectively reduced oxidative stress and apoptosis while promoting angiogenesis in transplanted ovarian tissues. The scaffold-based approach offers several advantages: it ensures high local melatonin concentrations at the graft site, minimizes systemic exposure, provides sustained release over the critical early post-transplantation period, and enables non-invasive application. Histological and immunohistochemical analyses revealed that scaffold-delivered melatonin preserved follicular morphology, increased vascular endothelial cell density, and reduced inflammatory cytokine levels compared to controls. Endocrine assessment showed accelerated recovery of estrous cycles, with treated animals achieving regular cyclicity earlier than untreated animals. The scaffold-based approach also demonstrated superior preservation of primordial and primary follicles, which represent the ovarian reserve and are crucial for long-term graft function^6^.

The collective evidence from our research group and others supports a multifaceted mechanism of melatonin action in ovarian tissue preservation. Melatonin preserves mitochondrial morphology and function, as evidenced by increased ATP levels and succinate dehydrogenase activity^2^. Beyond SOD2 the Nrf2/antioxidant response element signaling pathway, upregulating downstream antioxidant enzymes including heme oxygenase-1 and catalase^7^. The upregulation of GPx4 by melatonin is particularly significant for inhibiting ferroptosis, a recently recognized form of cell death characterized by iron-dependent lipid peroxidation^5^. Melatonin also promotes angiogenesis through multiple mechanisms, including increased vascular endothelial growth factor expression and enhanced SOD2 activity in endothelial cells, which is critical for graft survival^4^. Finally, the combined antioxidant and angiogenic effects of melatonin result in reduced apoptosis in granulosa cells, theca cells, and interstitial cells^4,7,8^.

Melatonin represents a compelling therapeutic adjunct in ovarian tissue preservation, acting through multiple complementary mechanisms—direct ROS scavenging, SOD2 and GPx upregulation, Nrf2 pathway activation, ferroptosis inhibition, and promotion of angiogenesis. Our systematic review has confirmed the robust antioxidant actions of melatonin across multiple animal models^1^, while our experimental studies have established its ability to increase SOD2 levels and improve graft function44, enhance GPx activity and antioxidant defense55, and achieve effective graft protection through scaffold-based delivery66. As ovarian tissue cryopreservation continues to expand in clinical practice, incorporating melatonin into preservation protocols—whether through systemic administration or innovative scaffold-based delivery—offers a safe, cost-effective strategy to maximize follicular survival and graft longevity. Future research should prioritize translational studies and clinical trials to establish evidence-based guidelines, ultimately improving fertility outcomes for young women facing gonadotoxic therapies.

## Data Availability

The datasets generated and/or analyzed during the current study are available from the corresponding author upon reasonable request.

## References

[1] Monteiro KKAC, Shiroma ME, Damous LL, Simões MJ, Simões RDS, Cipolla-Neto J (2024). Antioxidant actions of melatonin: a systematic review of animal studies. Antioxidants (Basel).

[2] Sun TC, Liu XC, Yang SH, Song LL, Zhou SJ, Deng SL (2020). Melatonin inhibits oxidative stress and apoptosis in cryopreserved ovarian tissues via Nrf2/HO-1 signaling pathway. Front Mol Biosci.

[3] Alkali IM, Colombo M, Luvoni GC (2024). Melatonin reduces oxidative stress and improves follicular morphology in feline (Felis catus) vitrified ovarian tissue. Theriogenology.

[4] Monteiro KKAC, Damous LL, Shiroma ME, Termini L, Cipolla-Neto J, Simões RDS (2024). Melatonin increases superoxide dismutase 2 (SOD2) levels and improves rat ovarian graft function after transplantation. J Ovarian Res.

[5] Monteiro KKAC, Damous LL, Shiroma ME, Turri JAO, Simões RDS, Simões MJ (2026). Melatonin enhances glutathione peroxidase activity and improves antioxidant defense in cryopreserved ovarian transplants: a rat model study. Antioxidants (Basel).

[6] Damous LL, Alves MS, Justino LG, Son ACH, Botelho NM, Shiroma ME (2026). Frozen-Thawed ovarian autografts treated with scaffold-based melatonin delivery in rats. Reprod Biol Endocrinol.

[7] Xie L, Wang S, Wu Y, Zhang X, Ding Y (2025). Melatonin protects intact rat ovarian transplantation via the MT1/Nrf2/ARE pathway. Cells.

[8] Shiroma ME, Damous LL, Cotrim FP, Roa CL, Cipolla-Neto J, Reiter RJ (2021). Pretreatment with melatonin improves ovarian tissue cryopreservation for transplantation. Reprod Biol Endocrinol.

